# Metabolic flexibility and resting autonomic function in active menopausal women

**DOI:** 10.1007/s00421-024-05568-2

**Published:** 2024-07-25

**Authors:** Jordi Monferrer-Marín, Ainoa Roldán, Jørn Wulff Helge, Cristina Blasco-Lafarga

**Affiliations:** 1 Sport Performance and Physical Fitness Research Group (UIRFIDE), Physical Education and Sports Department, University of Valencia, Valencia, Spain; 2 Department of Biomedical Sciences, Faculty of Health and Medical Sciences, University of Copenhagen, Copenhagen, Denmark

**Keywords:** Autonomic nervous system, Blood lactate, DFAα1, Fat oxidation, Heart rate variability

## Abstract

**Purpose:**

The present study aims to analyze the relationship between cardiac autonomic control at rest—i.e., baseline Heart Rate Variability (HRV)—and metabolic flexibility assessed by means of the FATox and CHOox oxidation rates at the intensities of maximum fat and carbohydrate oxidation (MFO and MCO, respectively).

**Methods:**

Twenty-four active over-60 women (66.8 ± 4.4 years) had their HRV assessed with 10 min recordings under resting conditions, and this was analyzed with Kubios Scientific software. After this, an incremental submaximal cycling test, starting at 30 watts, with increments of 10 watts every 3 min 15 s was performed. FATox and CHOox were calculated in the last 60 s at each step, using Frayn’s equation. MFO and MCO were further obtained.

**Results:**

Nonlinear SampEn and 1-DFAα1 (Detrending Fluctuation Analysis score) at rest were both moderate and significantly (*p* < 0.05) related to FATox (*r* = 0.43, *r* = −0.40) and CHOox (*r* = −0.59, *r* = 0.41), as well as RER (*r* = −0.43, *r* = 0.43) at FATmax intensity. At the MCO intensity, no association was observed between HRV and oxidation rates. However, DFAα1 (*r* = −0.63, *p* < 0.05), the frequency ratio LF/HF (*r* = −0.63, *p* < 0.05), and the Poincaré ratio SD1/SD2 (*r* = 0.48, *p* < 0.05) were correlated with blood lactate concentration.

**Conclusion:**

These results support the *autonomic resources hypothesis*, suggesting that better autonomic function at rest is related to enhanced metabolic flexibility in postmenopausal women. They also underpin a comprehensive analysis of cardiovascular-autonomic health with aging. The results imply that non-linear DFAα1 and SampEn are appropriate to analyze this association in health of the aging cardiovascular-autonomic system.

**Graphical Abstract:**

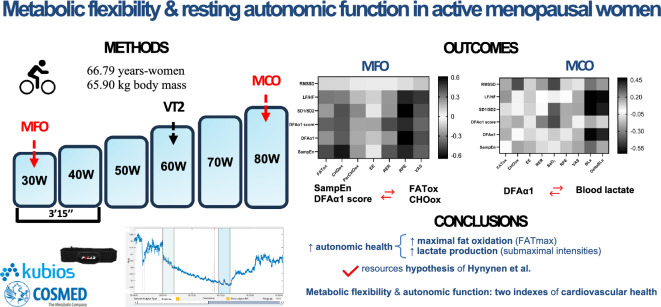

## Introduction

In recent decades, due to the increased incidence of cardiovascular disease, the study of cardiac activity and the involvement of the autonomic nervous system (ANS) function has become a subject of significant interest. Specifically, heart rate variability (HRV) analysis plays a key role in studying the reflex signals with origin in the brain, mediated by the sympathetic and vagus nerves, which innervate the sinoatrial node (Shaffer and Ginsberg 2017). This analysis serves as a non-invasive marker of the ANS function (Blasco-Lafarga et al. 2013). It describes the efficiency of neural feedback mechanisms and indicates an individuals’ state of health and/or their ability to mobilize physiological resources to respond appropriately to energy demands (Porges 2009). Indeed, the so-called *vagal brake* contributes to the modulation of cardiac output to adjust metabolic resources and support adaptive behaviors, with an immediate withdrawal and reengagement (inhibition and disinhibition) of vagal tone in response to physical and mental stimuli, including metabolically demanding states, such as exercise, stress, attention, and information processing (Porges 2007). Better cardiac autonomic control in the resting state is thus related to improved mental and physical health (Porges 2009) and performance (Blasco-Lafarga et al. 2013; Hynynen et al. 2008).

Similarly, major interest has been focused on metabolic flexibility, or rather its dysfunction, the metabolic inflexibility (Frandsen et al. 2021), the latter being an impaired ability to switch energy substrates in response to physiological demands (Galgani et al. 2008). This dysfunction (inflexibility) is accompanied by a downward and leftward shift of the energy substrate oxidation curves, resulting in lower fat oxidation at FATmax, and an increase in the rate of carbohydrate oxidation (Monferrer-Marín et al. 2022). Metabolic flexibility (or inflexibility) is linked to mitochondrial function, but it has an impact beyond the metabolic effect, as it directly associates with cardiovascular health (Wang et al. 2023). Specifically, improvements in both mitochondrial function and metabolic flexibility are associated with better cardiovascular health by reducing the risk of cardiovascular disease, decreasing ApoB/ApoAI ratios, thus improving the balance between atherosclerotic and anti-atherosclerotic cholesterol lipoproteins (Wang et al. 2023).

Given the cardiovascular link between autonomic function and metabolism, as well as the close relationship of metabolic inflexibility and autonomic dysfunction with the cardiovascular disease (Lee et al. 2022; Wang et al. 2023), it is interesting to investigate the relationship between these two cardiovascular aspects. Also of importance, both cardiovascular capacities are affected by sex differences and influenced by age-related impairment, so they may be altered in older women.

Autonomic behavior is influenced by sex (Lee et al. 2022), and women exhibit lower total power in spectral density in the cardiac signal with higher mean heart rates (Lee et al. 2022). Nonetheless, older women display a reduced low- and very low-frequency, and higher power in the high-frequency band compared to men (Lee et al. 2022). Increased vagal activity, which is positively associated with blood estrogen levels, likely explains for these frequency band differences. However, menopause leads to a significant reduction in cardiac vagal activity, resulting in increased sympathetic control in older women (von Holzen et al. 2016). Moreover, postmenopausal women, regardless of hormonal decline, experience a gradual reduction in overall autonomic input fluctuations to the heart due to the physiological aging process, and this further impairs the vagal index and contributes to sympathetic hyperactivity compared to their premenopausal counterparts (Shiels et al. 2019).

Other age-related alterations associated with declining estrogen levels include lower mitochondrial respiration and an impairment of fission–fusion dynamics (Yoh et al. 2023), and these dysfunctions contribute to the above-mentioned metabolic inflexibility (Frandsen et al. 2021). Older women display lower FATox which are not compensated by higher CHOox due to their low-energy expenditure, and this limits high muscle power already at submaximal intensities, despite reaching RER > 1 (Monferrer-Marín et al. 2022). Importantly, postmenopausal women who maintain high or sustained muscle power values exhibit better fat oxidation capacity at FATmax workloads, indicating a protective effect of physical exercise against metabolic inflexibility (Blasco-Lafarga et al. 2022; Galgani et al. 2008). The role of exercise in preserving metabolic flexibility and autonomic function may be of paramount importance in this population.

The present study aims to analyze the relationship between cardiac autonomic control at rest—measured by baseline HRV—and metabolic flexibility, assessed through FATox and CHOox rates at maximum fat and carbohydrate oxidation intensities (MFO and MCO, respectively). There is lack of studies on women regarding metabolic flexibility and autonomic health, and studies in old women are nonexisting. A significant relationship between autonomic function and metabolic flexibility, in a group of active postmenopausal women during a submaximal incremental test, would reinforce the autonomic resources hypothesis of Hynynen et al. (Hynynen et al. 2008).

## Methods

### Participants and experimental procedure

**Fig. 1:**
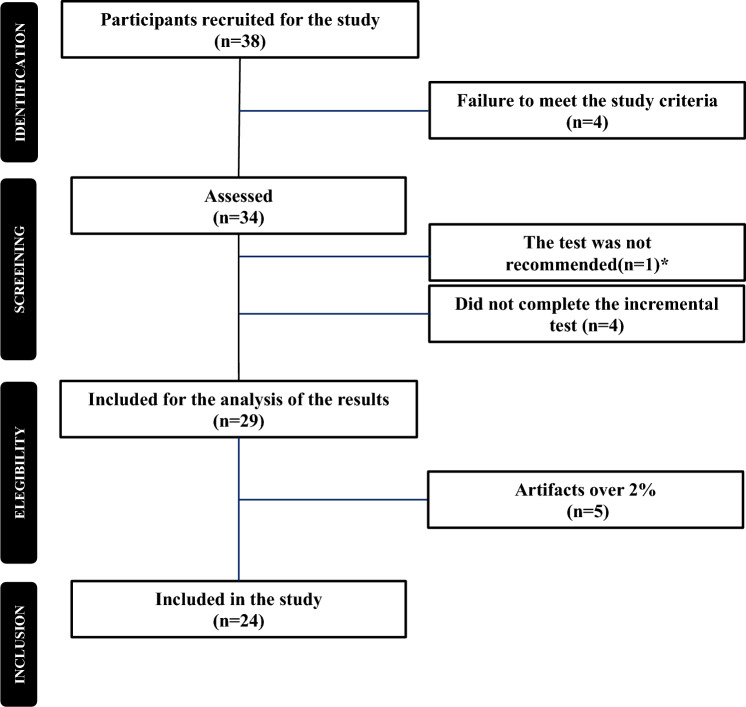
Flowchart of participants. *On the day of the test, the participant showed an isolated diastolic hypertension (> 90 mmHg), which prevented the protocol from being carried out safely

Thirty-eight active women volunteered to participate in the study. Twenty four of them completed the study as indicated in the flowchart (Fig. 1). This number exceeded the required 21 participants necessary to achieve a coefficient of determination greater than 0.3, with an alpha value of 0.05 and a power of 80%, as calculated using the G*Power software (version 3.1.9.6; Heinrich-Heine-Universität Düsseldorf, Düsseldorf, Germany). Inclusion criteria for participants were as follows: (1) postmenopausal women over 60 years old, (2) moderate physical activity level according to the International Physical Activity Questionnaire (at least a total of 600 METs), and (3) no medical contraindications to physical activity according to the Physical Activity Readiness Questionnaire. Exclusion criteria included: (1) diagnosed with T2DM or pre-diabetic, (2) use of medications (e.g., beta-blockers) that limit or affect physical activity, and (3) being on hormone replacement therapy or estrogen treatment of any kind. Blood pressure was also considered, consulting with health personnel in cases of values outside the normative range, i.e., exclusion from the study was considered in case of hypertension on the day of the test.

All participants were provided with information about the protocol, including its potential risks and benefits, and all signed the written informed consent form. The experimental procedure adhered to the principles of the Declaration of Helsinki and received approval from the local Ethics Committee (H105715353921).

Participants were asked to come without having performed intense exercise 24 h before the test and without altering their usual diet, maintaining their macronutrient composition and energy content, except for the pre-test dinner (> 50% kcal carbohydrate intake). They maintained at least 2 h of food fasting, with a recommendation of overnight abstinence, avoiding caffeine consumption, as proposed by San-Millan and Brooks (2017).

### Submaximal graded test, metabolic flexibility, and substrates’ evolution calculation

**Fig. 2:**
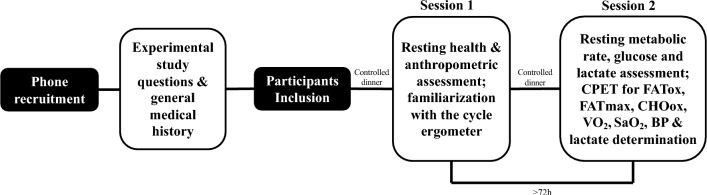
Experimental procedure. *CPET* cardiopulmonary exercise testing, *FATox* fat oxidation, *FATmax* intensity at which *FATox* is reached, *CHOox* carbohydrate oxidation, *VO*_*2*_ oxygen consumption, *SaO*_*2*_ oxygen saturation, *BP* blood pressure

As shown in Fig. 2, and following the approach proposed by San-Millán and Brooks (2017), on the testing day, metabolic flexibility was measured using FATox and CHOox oxidation rates, both calculated in absolute (g/min) and normalized to fat-free mass (FFM; mg/min/kg FFM) values (Amaro-Gahete et al. 2019), obtained by bioimpedance (Tanita DC-430 MA S; Tokyo, Japan).

In the incremental submaximal cycling test (Monferrer-Marín et al. 2022), as per the methodology described by these authors, the protocol commenced at 30 W, with increments of 10 W every 3 min and 15 s monitored by the smart roller Saris and the Rouvy application (VirtualTraining, Vimperk, Czech Republic). Metabolic flexibility calculations were based on the last 60 s (Amaro-Gahete et al. 2018) at each intensity level using Frayn’s equation (Frayn 1983), allowing to determine the maximal fat oxidation (MFO) point, reached at FATmax intensity, and maximal carbohydrate point (MCO), reached at the end of the test. VO_2_ and VCO_2_ were measured by indirect calorimetry using the K4b2 metabolic chart (Cosmed, Rome, Italy). The gas analysers were carefully calibrated with an automated volume calibration and with a gas mixture recommended by the manufacturer prior to the start of each test. Considering the growing relevance of carbohydrate oxidation (Blasco-Lafarga et al. 2022), the analysis also included the percentage of carbohydrate oxidation at the point of maximal fat oxidation, relative to its peak, and referred to this variable as PerCHOox.

Before starting the incremental test, as well as at 3 and 5 min after its completion, capillary blood samples were collected from the index finger of the left hand to measure blood lactate levels using the Lactate Scout device (SensLab GmbH, Leipzig, Germany). Additionally, the changes in blood lactate levels (DeltaBLa; mmol/L) were calculated. See Monferrer-Marín et al. (2022) for more details about the experimental procedure, data collection, and analysis.

During the test, the rate perceived effort (RPE) of Borg was registered, along with the visual analog scale of pain (VAS) (Hicks et al. 2001). Both scales ranges from 0 to 10, with 0 representing no exertion and 10 indicating maximum exertion.

### Autonomic resources’ measurement and cardiac signal processing

On the session 1 (Fig. 2), RR data were recorded for 10 min by chest strap device Polar H10 (Polar Electro Oy, Kempele, Finland) for the analysis of HRV under resting conditions. The women were asked to remain comfortably seated with their heads tilted and eyes closed in a quiet room. RR recordings were subsequently exported from the Polar Sensor Logger app to the Kubios Scientific software (version 4.0.2; Biosignal Analysis and Medical Imaging Group, Department of Physics, University of Kuopio, Kuopio, Finland) for further analysis. Artifacts were identified and corrected (Lambda = 500) using Kubios’ “automatic method” (Tarvainen et al. 2014), with those that exceeded 2% excluded from the analysis (Gronwald et al. 2019). The 5 min RR windows that demonstrated the highest signal stability were selected, while the remaining recordings were discarded (Shaffer and Ginsberg 2017).

In the context of linear HRV time-domain measures during resting conditions, the root mean square of successive differences between normal beats (RMSSD, in milliseconds) was selected, as it is the linear variable by reference, reflecting the vagal reactivation (Laborde et al. 2024). Additionally, in a frequency-domain analysis, the ratio of low frequency (LF, 0.04–0.15 Hz) to high frequency (HF, 0.15–0.4 Hz) (LF/HF) was calculated using the fast Fourier transform analysis (Shaffer and Ginsberg 2017), given its relationship with non-linear indices (Doret et al. 2015).

Regarding, the non-linear dynamics of cardiac variability: geometric, fractal, and entropy methods were assessed.

For geometric non-linear methodology, the Poincaré diagram was used to determine the ratio (SD1/SD2) between the width of the ellipse (parameter SD1 in milliseconds) and the length of the ellipse (parameter SD2 in milliseconds). This ratio reflects the dispersion of points perpendicular to the line of identity and is associated with rapid beat-to-beat variations, which describe parasympathetic activation (Kim et al. 2017). Fractal and entropy methods were applied to analyze the unpredictability of a time-series and capture the complexity of autonomic function regulation (Shaffer and Ginsberg 2017). These measures are particularly sensitive to rapid responses and signal instability under conditions of physical exercise (Blasco-Lafarga et al. 2017).

The detrended fluctuation analysis (DFA) algorithm was employed to explore correlations between RR intervals at various time scales (Blasco-Lafarga et al. 2017). This method is useful for assessing long-term autocorrelation in non-stationary time-series (Zimatore et al. 2022). It provides insights into cardiac system fluctuations across multiple time scales, with lower self-similarity indicating a more randomly structured, less adaptive, and less flexible system (Pham et al. 2021). Specifically, the short-term or α1 correlations derived from DFA reflect the vagal activity (Shaffer and Ginsberg 2017). The window size for DFAα1 analysis was set to encompass 4–16 beats (Peng et al. 1995). To eliminate bidirectional scaling properties and prevent masking enhancement in the data, the score relative to the theoretical baseline value of 1 in DFAα1 was used (│1-α1│), as proposed by Millar et al. (2009).

Furthermore, sample entropy (SampEn) was employed to gauge the complexity of the time-series under these conditions, offering insights into cardiovascular functionality (Kumar et al. 2019).

To gain insight into the MFO and MCO intensities, and to ensure cardiac signal stability, the final 2 min of the steps in the test were retained, with the initial 1 min and 15 s (adaptation period to increased intensity) excluded (Blasco-Lafarga et al. 2017; Gronwald et al. 2019). Given the short recording length during the incremental test, only HRV variables suitable for measurements of 2 min or less were analyzed. This included RMSSD, SD1/SD2 ratio, and DFAα1 (Gronwald et al. 2019).

### Statistical analysis

Statistical analysis was conducted using the Statistical Package for Social Sciences (SPSS, version 25.0, IBM SPSS Statistics, IBM Corporation). Data were presented as mean and standard deviation (SD). The Shapiro–Wilk test was utilized to assess the normality of the data. To compare MFO and MCO, the Wilcoxon test was performed. Bivariate Spearman correlations were employed to investigate the relationships between HRV variables at rest and the primary markers of metabolic flexibility during the incremental test. These associations were visualized in a heatmap displaying the correlation coefficients between the variables under comparison (refer to Figs. 5 and 6). We also generated scatter plots to illustrate the linear HRV variables alongside the non-linear HRV variables with FATox and BLapeak at MFO and MCO, respectively (Fig. 5). All figures were created using GraphPad Prism® 10 (version 10.01, GraphPad Software, Inc., La Jolla, California, USA).

The *p* value of significance was *p* < 0.05. Sullivan and Feinn’s (2012) classification was used for both the Spearman’s *r* correlation: small (*r* = 0.20), medium (*r* = 0.50), or large (*r* = 0.80); and the coefficient of determination (*R*^2^): small (*R*^2^ = 0.04), medium (*R*^2^ = 0.25), or large (*R*^2^ = 0.64).

## Results

Table 1 summarizes the main descriptive data of the final sample (*n* = 24), consisting of women aged between 64 and 68 years, with normal weight for their age, and preserved muscle mass.

**Table 1:** Anthropometric, cardiovascular and heart rate variability data [mean (SD)] at rest (*n* = 24)

Anthropometric and cardiovascular baseline variables		Lactate and heart rate variability variables	
Age (years)	67 (4)	HR (bpm)	70 (10)
Height (cm)	158.1 (6.5)	SaO2 (%)	96 (1)
Body mass (kg)	65.90 (10.87)	RMSSD (ms)	16.63 (7.88)
BMI (kg/m^2^)	27.03 (3.71)	Ratio LF/HF	3.57 (3.67)
Fat mass (%)	34.79 (4.19)	Ratio SD1/SD2	0.47 (0.13)
FFM (kg)	41.64 (5.95)	DFAα1 score	0.25 (0.18)
Muscle mass (kg)	39.47 (4.6)	DFAα1	1.17 (0.26)
SBP (mmHg)	125 (15)	SampEn	1.64 (0.25)
DBP (mmHg)	79 (9)	BLa (mmol/L)	1.6 (0.9)

*BMI* body mass index, *FFM* fat-free mass, *SBP* systolic blood pressure, *DBP* diastolic blood pressure, *HR* heart rate, *SaO2* oxygen saturation, *RMSSD* root-mean-square root of successive differences between normal beats, *LF/HF* ratio between low and high frequencies, *SD1/SD2* ratio between both axes of the Poincaré plot, *DFAα1_score* [1-DFA α1], *DFAα1* short-term detrended fluctuation analysis, *SampEn* sample entropy, *BLa* blood lactate

MFO and MCO intensities in the test show differences across all outcomes, as hypothesized (Table 2). More specifically, HR, CHOox, power, RER, energy expenditure, and DFAα1 score increased from MFO to MCO, and as expected, there was a decrease in FATox, RMSSD, DFAα1, and intensity, and the lactate value was 6.9 (3.4) mmol/L.

**Table 2:** Physiological responses at MFO and MCO intensities (*n* = 24). Data are [mean (SD)]

	MFO	MCO	*P* value
RPE	0.73 (1.31)	4.68 (2.14)	< 0.001
VAS	0.23 (0.69)	1.18 (2.11)	0.009
FATox (mg/min/kg)	4.01 (2.11)	0.70 (1.03)	< 0.001
CHOox (mg/min/kg)	15.70 (6.94)	45.46 (17.60)	< 0.001
Power (W)	40.00 (10.24)	81.82 (13.68)	< 0.001
RER	0.87 (0.07)	1.07 (0.12)	< 0.001
EE (kcal/min)	3.74 (1.10)	6.06 (2.12)	< 0.001
RMSSD (ms)	7.03 (3.71)	2.99 (1.40)	< 0.001
DFAα1 score	0.28 (0.20)	0.47 (0.24)	0.001
DFAα1	1.06 (0.34)	0.57 (0.31)	< 0.001

Mean (standard deviation)

*MFO* maximal fat oxidation, *MCO* maximal carbohydrate oxidation, *RPE* rate of perceived exertion, *VAS* visual analog pain scale, *FATox* fat oxidation, *CHOox* carbohydrate oxidation, *RER* respiratory exchange ratio, *EE* energy expenditure, *RMSSD* root-mean-square root of successive differences between normal beats, *DFAα1* short-term detrended fluctuation analysis, *DFAα1 score* [1-DFA α1]

At rest, DFAα1 showed a significant, large, and positive correlation with LF/HF (*r* = 0.92; *p* < 0.05) and with its score (*r* = 0.69; *p* < 0.05), and a negative and large correlation with SD1/SD2 (*r* = −0.88; *p* < 0.05) and SampEn (*r* = −0.74; *p* < 0.05), with no relationship with RMSSD (*p* = 0.387).

**Fig. 3:**
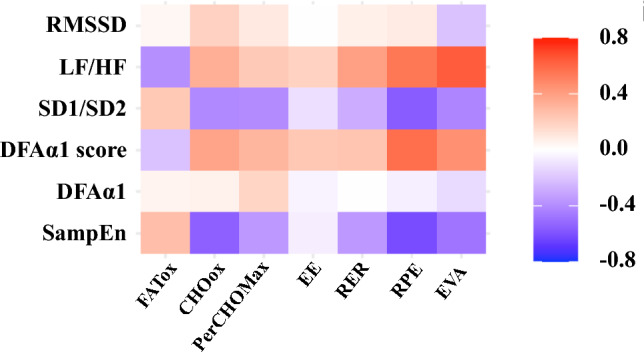
Spearman correlations between autonomic function and metabolic flexibility variables in MFO at FATmax intensity. *RMSSD* root-mean-square root of successive differences between normal beats, *LF/HF* ratio between low and high frequencies, *SD1/SD2* ratio between both axes of the Poincaré plot, *DFAα1* short-term detrended fluctuation analysis, *DFAα1_score* [1-DFA α1], *SampEn* sample entropy, *FATox* fat oxidation, *CHOox* carbohydrate oxidation, *PerCHOox* percentage of CHOox over its peak value, *EE* energy expenditure, *RER* respiratory exchange ratio, *RPE* rate of perceived exertion, *VAS* visual analog pain scale

For the relationship between metabolic flexibility and autonomic resources at rest, there were several noteworthy associations between many of the biomarkers at the point of MFO (Fig. 3). SampEn displayed a significant, moderate negative correlation with RER (*r* = −0.43; *p* < 0.05) and both energy substrates: CHOox (*r* = −0.59; *p* < 0.05) and FATox (*r* = 0.43; *p* < 0.05). While DFAα1 did not exhibit significant associations with energy substrates, there were significant correlations with the DFAα1 score. These correlations were moderately negative with FATox (*r* = −0.40; *p* < 0.05) and positive with RER (*r* = 0.43; *p* = 0.037) and CHOox (*r* = 0.41; *p* < 0.05). CHOox is also significantly and negatively associated with SD1/SD2 ratio (*r* = −0.44; *p* < 0.05). Furthermore, the variables RPE and VAS demonstrated significant, moderate positive associations with the LF/HF ratio (RPE: *r* = 0.53, *p* = 0.008; VAS: *r* = 0.48, *p* = 0 < 0.05), DFAα1 (RPE: *r* = 0.61, *p* < 0.05; VAS: *r* = 0.42, *p* < 0.05), and its score only with RPE (*r* = 0.61, *p* < 0.05). Additionally, these variables displayed significant, moderate negative associations with the SD1/SD2 ratio (RPE: *r* = −0.65, *p* < 0.05; VAS: *r* = −0.44, *p* < 0.05) and SampEn but only with RPE in their case (*r* = −0.55, *p* = 0.005).

**Fig. 4:**
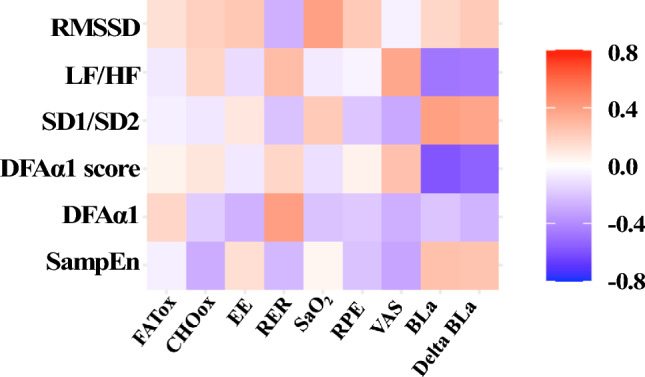
Spearman correlations between autonomic function and metabolic flexibility variables in MCO. *RMSSD* root-mean-square root of successive differences between normal beats, *LF/HF* ratio between low and high frequencies, *SD1/SD2* ratio between both axes of the Poincaré plot, *DFAα1* short-term detrended fluctuation analysis, *DFAα1_score* [1-DFA α1], *SampEn* sample entropy, *FATox* fat oxidation, *CHOox* carbohydrate oxidation, *EE* energy expenditure, *RER* respiratory exchange ratio, *SaO*_*2*_ oxygen saturation, *RPE* rate of perceived exertion, *VAS* visual analog pain scale, *BLa* blood lactate

Regarding the MCO point (Fig. 4), no correlations were observed between cardiac variability at rest and the main variables of metabolic flexibility. However, blood lactate at this time-point and its delta with respect to the pre-test were medium, negative, and significantly associated with the LF/HF ratio (BLa: *r* = −0.63, *p* < 0.05; DeltaBLa: *r* = −0.61, *p* < 0.05), and DFAα1 (BLa: *r* = −0.63, *p* < 0.05; DeltaBLa: *r* = −0.54, *p* < 0.05). The SD1/SD2 ratio at rest is positively associated with blood lactate at MCO (BLa: *r* = 0.48, *p* < 0.05; DeltaBLa: *r* = 0.43, *p* < 0.05).

**Fig. 5:**
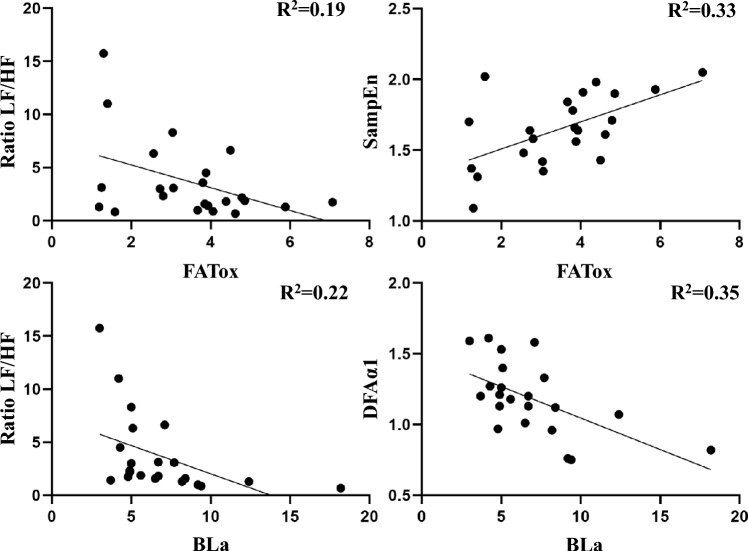
Scatter plots between linear (left column) and non-linear (right column) variables in MFO (top row) and MCO (bottom row). *LF/HF* ratio between low and high frequencies, *DFAα1* short-term detrended fluctuation analysis, *SampEn* sample entropy, *FATox* fat oxidation, *BLa* blood lactate

Finally, Fig. 5 shows the scatter plot of linear variables such as the LF/HF ratio (left column) and non-linear such as DFAα1 and SampEn (right column) at rest, with the values of FATox as an indicator of relevance in MFO (top row) and those of BLa as an MCO marker (bottom row) to support the study of associations. The coefficient of determination shows explained variability between SampEn and FATox (*R*^2^ = 0.33) and with DFAα1 and BLa (*R*^2^ = 0.35), medium grade in both cases, which is lower, but equally medium, with the LF/HF ratio and the same elements, respectively (*R*^2^ = 0.19; *R*^2^ = 0.22).

## Discussion

The main novel finding was a relationship between autonomic resources under resting conditions and metabolic flexibility variables at both the MFO and MCO intensities in older women. The active lifestyle (> 600 METs) of these women may have played a role in the maintenance of their healthy autonomic-cardiovascular capacity despite reduced muscle power and impaired oxidation rates at MFO and MCO compared to a younger population (Blasco-Lafarga et al. 2022).

As expected, the associated HRV variables differed between these two intensities. Interestingly, in FATmax intensity, both SampEn and the DFAα1 score emerged as the key autonomic resources markers due to our association with both energy substrates (FATox and CHOox). In MCO, no association was observed with FATox and CHOox; nonetheless, DFAα1 correlated with blood lactate, other substrate with metabolic relevance, underlying the link between non-linear variables and metabolic markers.

The autonomic resources observed in the resting sitting position displayed lower values than expected in terms of RMSSD, and SampEn, while the LF/HF ratio and DFAα1 exhibited higher values, when compared to a population with age and body composition similarities (Martínez Navarro 2014). These findings could potentially be attributed to age-associated sympathetic activation (Shiels et al. 2019). When compared to a similar population (Martínez Navarro 2014), albeit measured in the supine position, these values also suggest a higher level of sympathetic activity within our sample.

These disagreements with the above-mentioned literature could be due to differences in recording, given the resting conditions of our sample, as women had to travel to the laboratory, as opposed to baseline conditions. For example, the RR recording position in our study was seated, which differs from the supine position (Molina et al. 2016). Furthermore, the nutritional status of the participants could have an influence, given that the participants in our study were not overnight fasted. The nutritional status was intentional and aimed at ensuring the reproducibility of the test, as pointed out by Amaro-Gahete et al. (2018) and to avoid any effort limitation due to low substrate availability, with a previous breakfast at least 2 h before, and a controlled dinner to minimize the nutritional influence.

For MFO, the main associations between autonomic resources at rest and metabolic flexibility were primarily between the SampEn and the DFAα1 score, as HRV variables, and RER, as well as energy substrates (FATox and CHOox), as metabolic flexibility parameters. These findings underscore that women who show a better vagal activity at rest, as represented by SampEn and DFAα1 scores, also show a higher fat oxidation (i.e., MFO). This interaction between the nervous system and metabolic flexibility may be explained by the multisystemic interaction within the liver–brain–adipose–neural tissue axis (Imai and Katagiri 2022). These authors emphasize the significant role of the nervous system in influencing liver function concerning the mobilization and utilization of fat and carbohydrates (Imai and Katagiri 2022). The nervous system regulates both glucose and fatty acids (Imai and Katagiri 2022), given the direct action of the vagal nerve on the liver depending on blood glucose concentrations, or substrate availability. The vagal nerve also has the ability to modulate insulin, glycogen, and leptin concentrations to maintain glucose homeostasis (Matsubara et al. 2022).

Therefore, in situations of moderate intensity, such as FATmax—the intensity at which MFO is reached—the better the autonomic resources at rest (i.e., better cardiovascular health) the higher the energy derived from fats may be. If so, those women would benefit from the possibility to increase energy expenditure and the utilization of carbohydrates as a substrate at higher intensities (Monferrer-Marín et al. 2022), a regulatory process in which the vagal nerve also contributes (Guarino et al. 2017).

Consequently, maintaining resting sympathovagal balance and a well-functioning vagal activity lead to better metabolic flexibility in MFO intensities, which could be related to the effective communication between the liver and the brain via the sympathetic (afferent) and vagus (efferent) nerves (Matsubara et al. 2022), pointing to these non-linear variables as qualified descriptors of cardiovascular and metabolic regulation and control systems (Kaufmann et al. 2023). This interaction and maintenance of balance would enhance the ability to effectively modulate energy substrate availability.

Other HRV markers, such as the SD1/SD2 ratio, exhibit associations exclusively with carbohydrate oxidation, highlighting the significant role of the vagus nerve in metabolic control (Imai and Katagiri 2022). In parasympathetic predominance, athletes display a better oxidative performance due to their lower sympathetic and glycolytic involvement, which is related to the promotion of glycogen storage and the non-activation of insulin mechanisms in parasympathetic-dominant situations (Matsubara et al. 2022). The delayed activation of the sympathetic nervous system may lead to a delayed glucose release and a subsequent decrease in carbohydrate utilization at intensities such as FATmax, as enhanced fat oxidation ensures energy supply at this point. This implies that, despite the early sympathetic activation at the start of the test and the anticipation, or even predominance, of CHOox in the elderly population, those women with a higher sympathovagal balance (i.e., higher SD1/SD2 ratio) have a lower CHOox at MFO because of the high utilization of fat as a substrate at this point (Frandsen et al. 2019). This capacity would allow these older women to be able to respond, by increasing CHOox, to greater energy demands at higher intensities. It reinforces the relevance of analyzing the CHOox (as well as a low PerCHOox) along the test, as noted in recent studies, in which this variable has been shown as the only variable associated with overall metabolic efficiency (Blasco-Lafarga et al. 2022).

At the intensity of MCO, in contrast to the peak fat oxidation, FATmax, there are no associations observed between any of the autonomic resources and the ratios of fat and carbohydrate oxidation or RER. However, three biomarkers are associated with blood lactate levels (BLa).

Noteworthy, the SD1/SD2 ratio was the only HRV variable associated with metabolic flexibility variables at both intensities of the test. Again, it highlights the relevance of this ratio in glycolytic performance. Both DFAα1 and LF/HF ratio associations with BLa further reinforce this pattern, with the higher associations with blood lactate. These associations indicate that a vagal activity at rest not only coincides with reduced carbohydrate oxidation during MFO, but also with increased lactate oxidation during MCO. This occurs despite the delayed glycolytic predominance or a slower parasympathetic withdrawal at moderate intensities such as FATmax, since when withdrawal occurs, the energy demands required by CHOox increase to a greater extent, reaching higher values at MCO. Indeed, the lactate association with DFAα1 was expected, since this variable, which encompasses interactions at the electrophysiological, hemodynamic, humoral, and cerebral levels (Blasco-Lafarga et al. 2017), is a key molecule in peripheral-central signaling and information exchange (Brooks 2018). Given the vagus nerve’s influence on lactate in the liver, it seems obvious that a significant part of lactate was used to synthesize glucose through the Cori cycle (Imai and Katagiri 2022).

All these findings suggest a synchronization between the autonomic nervous system and substrates utilization, aligning with Porges (2007) or Matsubara et al. (2022). These latter studies underscore the significant importance of the autonomic resources at rest for subsequent physical performance, at least at submaximal intensities. In our sample, with maintained metabolic flexibility despite the lower energy expenditure (Blasco-Lafarga et al. 2022), this higher physical performance associated with autonomic resources is observed as higher peak fat oxidation at FATmax, together with higher lactate production at submaximal intensities, which in turn is associated with greater total power (Monferrer-Marín et al. 2022). This connection between metabolic flexibility and autonomic health reinforces the autonomic resources hypothesis of Hynynen et al. (Hynynen et al. 2008). This relationship between metabolic flexibility and autonomic health would be explained by the close relationship between heart and fat oxidation (Actis Dato et al. 2024), postulating both cardiovascular abilities as relevant to cardiovascular studies, which reinforces the need of a comprehensive analysis of cardiovascular-autonomic health.

In line with the hypothesis of Blasco-Lafarga et al. (2013), the results indicate a higher variability explained by the non-linear variables both in MFO, with SampEn being more descriptive of FATox than the LF/HF ratio, and even for MCO with lactate. At this point, the LF/HF ratio shows a lower explained variability (22% vs. 35%) than DFAα1, despite its strong association. This may be explained by the ability of these non-linear variables to capture signals from the cardiovascular and metabolic regulatory systems (Shaffer and Ginsberg 2017).

This experimental design is not without limitations. It is important to note that this is a preliminary study with a small sample size, as well as it being composed mainly of female Nordic walking practitioners, which could limit the generalizability of the findings and their external validity. Furthermore, the study is based on indirect measurements of both the autonomic nervous system, using HRV, and the oxidation of energy substrates, using indirect calorimetry. Therefore, future studies should consider expanding the sample size, with more direct investigations of both aspects of cardiovascular health and simultaneously examining resting and different exercise conditions for both capacities.

## Conclusions

In summary, the results support that women with better cardiac autonomic control at rest show better metabolic flexibility during a submaximal incremental test. This finding confirms the associations between both cardiovascular capacities and suggests the need of a comprehensive assessment of cardiovascular health for study the aging impact. The vagal activity shows an association with fat and carbohydrate substrates. Future research should confirm the HRV as a key marker of the liver and vagal nerve interaction and its impact on metabolic flexibility.

Moreover, this outcome could improve the adjustment of training loads or exercise metabolic demands based on the resting ANS status (cardiac autonomic control). Future studies may focus in these HRV indexes to design more accurate and effective training programs in populations with special needs, such as postmenopausal adult women, allowing these tools to be used to improve cardiovascular health.

## Data Availability

The datasets generated and/or analyzed during the current study are not publicly available due to the conditions of the ethical approval provided by the Valencia University Human Research Ethics Committee. Notwithstanding, the anonymous data and analysis are available from the corresponding author on reasonable request.

## Abbreviations

ApoB/ApoAI: Ratio of apolipoprotein B and AI
BLa: Blood lactate
BMI: Body mass index
CHOox: Carbohydrate oxidation
DBP: Diastolic blood pressure
DFAα1: Detrended fluctuation analysis short term
EE: Energy expenditure
FATmax: Intensity at which MFO was reached
FATox: Fat oxidation
FFM: Fat-free mass
HRV: Heart rate variability
LF/HF: Ratio of low-frequency and high-frequency bands
MCO: Maximal carbohydrate oxidation intensity
METs: Metabolic equivalent
MFO: Maximal fat oxidation point
PerCHOox: Percentage of carbohydrate oxidation at FATmax
RER: Respiratory exchange ratio
RMSSD: Root mean square of successive differences
RPE: Rate of perceived exertion
RR: Time elapsed between two successive R waves of the QRS signal
SampEn: Sample entropy
SBP: Systolic blood pressure
SD1/SD2: Ratio of width to length of the ellipse of the Poincaré plot
T2DM: Type 2 diabetes mellitus
VAS: Visual analog scale
